# Supplementary material for: The representational hierarchy in human and artificial visual systems in the presence of object-scene regularities
Source: PLoS Comput Biol. 2023 Apr 28;19(4):e1011086. doi: 10.1371/journal.pcbi.1011086 (PMC10171658; doi:10.1371/journal.pcbi.1011086)
Supplement: S1 Fig — The main brain RSA analysis is replicated with alternative distance measures: (A) cross-validated Mahalanobis distance following [1] and (B) Euclidean distance. As for the main RSA, we tested 4 models: GIST, condition, domain, co-occurrence. Results confirm data analysis performed with 1-corr distance. Filled bars indicate significant values against baseline (p <0.005, corrected for n. or ROIs) calculated with pairwise t-tests across subjects (n = 19). (DOCX) [file pcbi.1011086.s001.docx]

**S1 Fig**


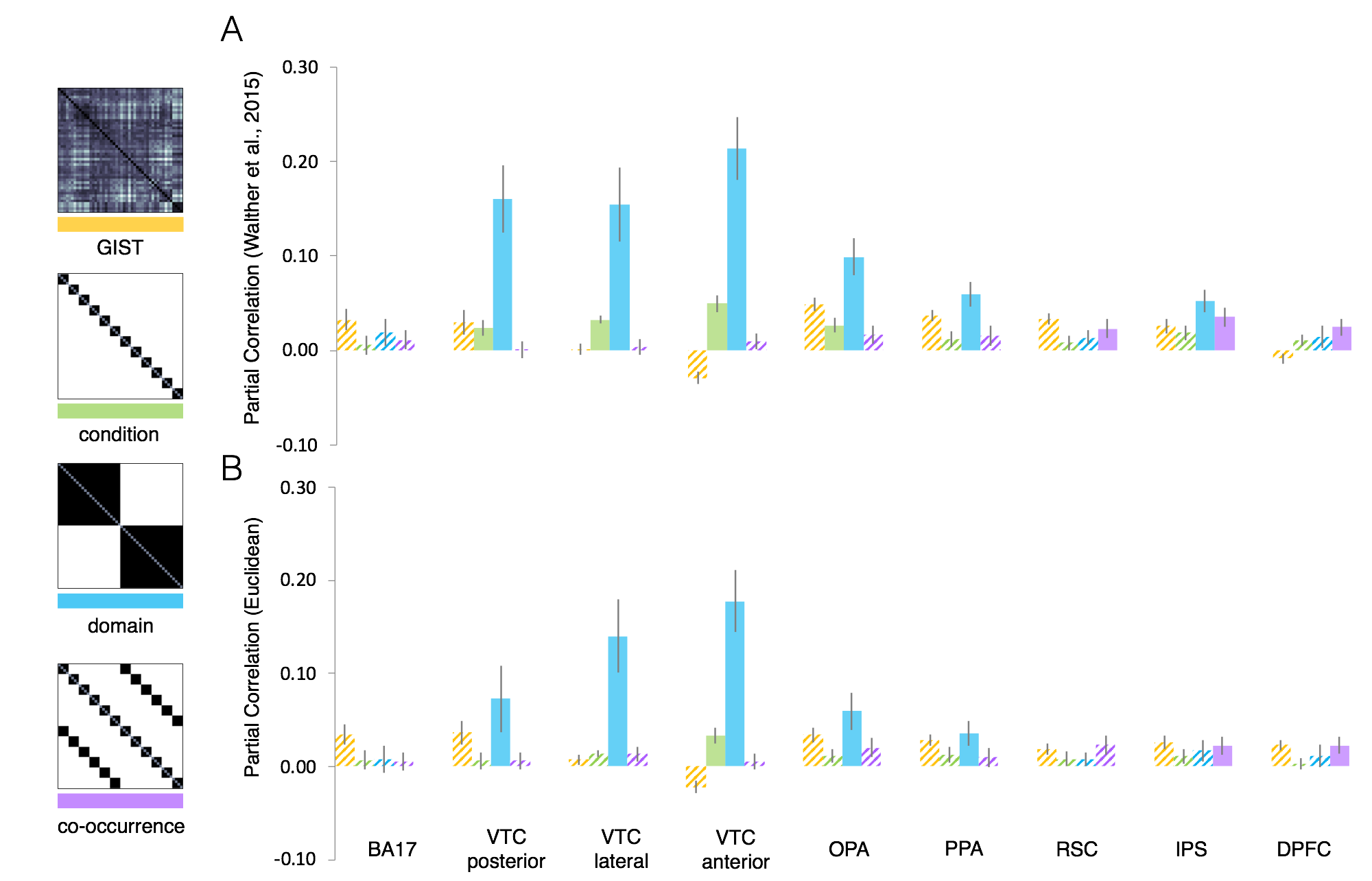


***S1 Fig. Dissimilarity matrices computed with alternative distance metrics.*** *The main brain RSA analysis is replicated with alternative distance measures: (A) cross-validated Mahalanobis distance following [1] and (B) Euclidean distance. As for the main RSA, we tested 4 models: GIST, condition, domain, co-occurrence. Results confirm data analysis performed with 1-corr distance. Filled bars indicate significant values against baseline (p <0.005, corrected for n. or ROIs) calculated with pairwise t-tests across subjects (n=19).*

**References**

1. Walther A, Nili H, Ejaz N, Alink A, Kriegeskorte N, Diedrichsen J. Reliability of dissimilarity measures for multi-voxel pattern analysis. NeuroImage. 2016;137:188-200. Epub 2015/12/29. doi: 10.1016/j.neuroimage.2015.12.012. PubMed PMID: 26707889.

2. Smith SM, Nichols TE. Threshold-free cluster enhancement: addressing problems of smoothing, threshold dependence and localisation in cluster inference. NeuroImage. 2009;44(1):83-98. doi: 10.1016/j.neuroimage.2008.03.061. PubMed PMID: 18501637.

3. Xia M, Wang J, He Y. BrainNet Viewer: a network visualization tool for human brain connectomics. PloS one. 2013;8(7):e68910. doi: 10.1371/journal.pone.0068910. PubMed PMID: 23861951; PubMed Central PMCID: PMC3701683.
