# Supplementary material for: The representational hierarchy in human and artificial visual systems in the presence of object-scene regularities
Source: PLoS Comput Biol. 2023 Apr 28;19(4):e1011086. doi: 10.1371/journal.pcbi.1011086 (PMC10171658; doi:10.1371/journal.pcbi.1011086)
Supplement: S2 Fig — (A) For each DCNNs, the RSA results show the degree of similarity between the representational space of individual layers in each brain area. (B) The random-effects whole-brain RSA results corrected with Threshold-Free Cluster Enhancement [TFCE; 2] are displayed separately for each layer of AlexNet against baseline [BrainNet Viewer; 3]. (DOCX) [file pcbi.1011086.s002.docx]

**S2 Fig**

*
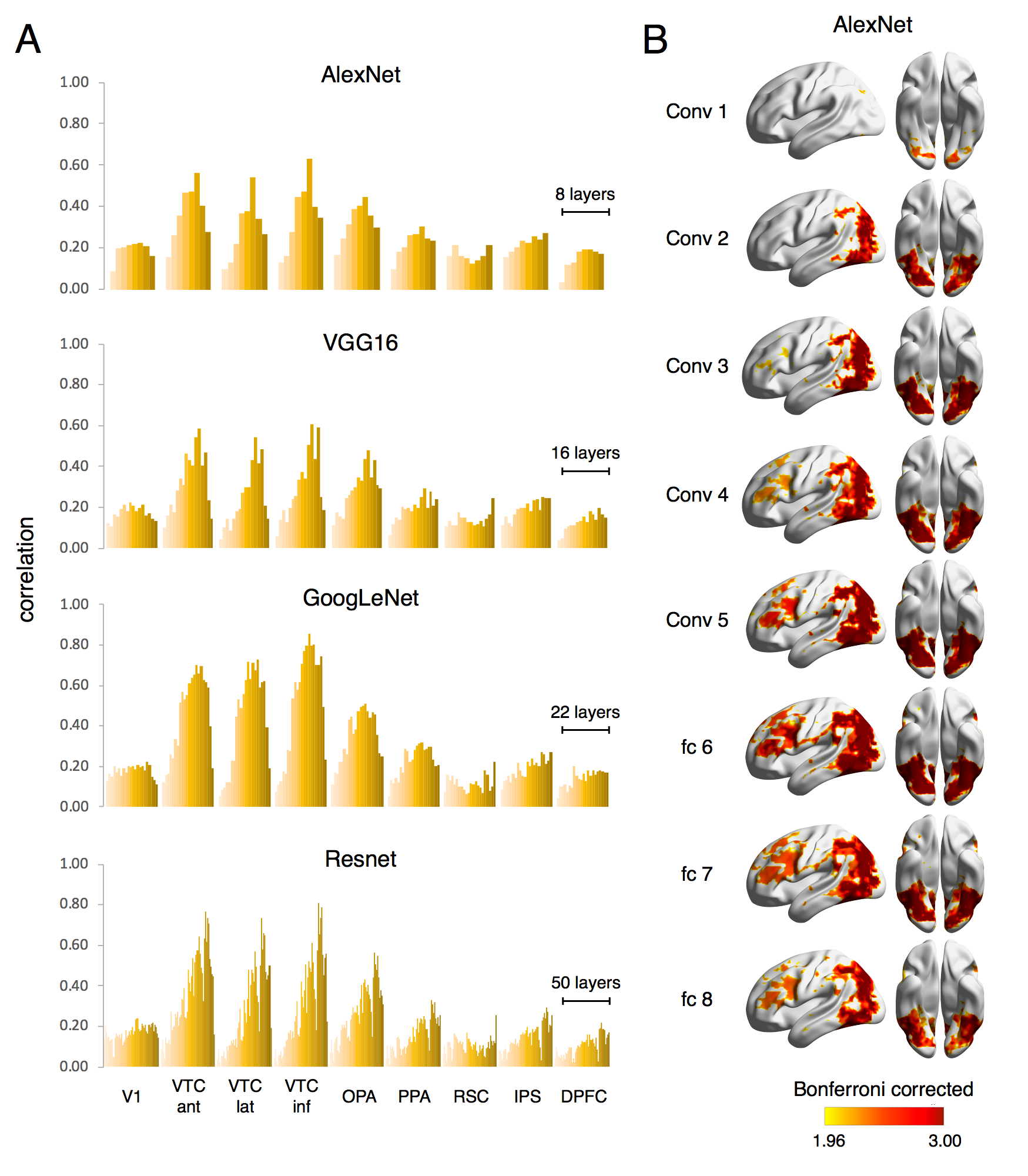
*

***S2 Fig. VTC representations are best captured by mid-level DCNNs layers.*** *(A) For each DCNNs, the RSA results show the degree of similarity between the representational space of individual layers in each brain area. (B) The random-effects whole-brain RSA results corrected with Threshold-Free Cluster Enhancement [TFCE; 2] are displayed separately for each layer of AlexNet against baseline [BrainNet Viewer; 3].*

**S2 Text**

Results from this analysis confirm results reported in Figure 3 showing that representations in ventral stream domain-specific areas (animal- and scene-selective areas) are best captured by mid-layers as opposed to the latest layers. Conversely, representational correspondence with frontoparietal areas does not differ between mid and top layers. This effect was observed regardless of DCNNs architecture.

**References**

1. Walther A, Nili H, Ejaz N, Alink A, Kriegeskorte N, Diedrichsen J. Reliability of dissimilarity measures for multi-voxel pattern analysis. NeuroImage. 2016;137:188-200. Epub 2015/12/29. doi: 10.1016/j.neuroimage.2015.12.012. PubMed PMID: 26707889.

2. Smith SM, Nichols TE. Threshold-free cluster enhancement: addressing problems of smoothing, threshold dependence and localisation in cluster inference. NeuroImage. 2009;44(1):83-98. doi: 10.1016/j.neuroimage.2008.03.061. PubMed PMID: 18501637.

3. Xia M, Wang J, He Y. BrainNet Viewer: a network visualization tool for human brain connectomics. PloS one. 2013;8(7):e68910. doi: 10.1371/journal.pone.0068910. PubMed PMID: 23861951; PubMed Central PMCID: PMC3701683.
