# Supplementary material for: The representational hierarchy in human and artificial visual systems in the presence of object-scene regularities
Source: PLoS Comput Biol. 2023 Apr 28;19(4):e1011086. doi: 10.1371/journal.pcbi.1011086 (PMC10171658; doi:10.1371/journal.pcbi.1011086)
Supplement: S3 Fig — The RSA results for the 4 models (GIST, condition, animacy continuum, navigational layout) are shown for group-averaged brain (left) data and DCNNs (right). The same DCNN architecture (GoogLeNet) was trained either on object recognition (ImageNet), or scene recognition (Scene 365). For comparison with the first RSA analysis (see Fig 3A), gray shaded areas indicate the network’s layers in which the domain model significantly outperformed the remaining models. Color-coded lines on top of bar/graphs indicate the network’s layers/ROIs where each model significantly outperformed the remaining models (p < 0.001) computed with pairwise permutations tests (10000 randomizations of stimulus labels). (DOCX) [file pcbi.1011086.s003.docx]

**S3 Fig**


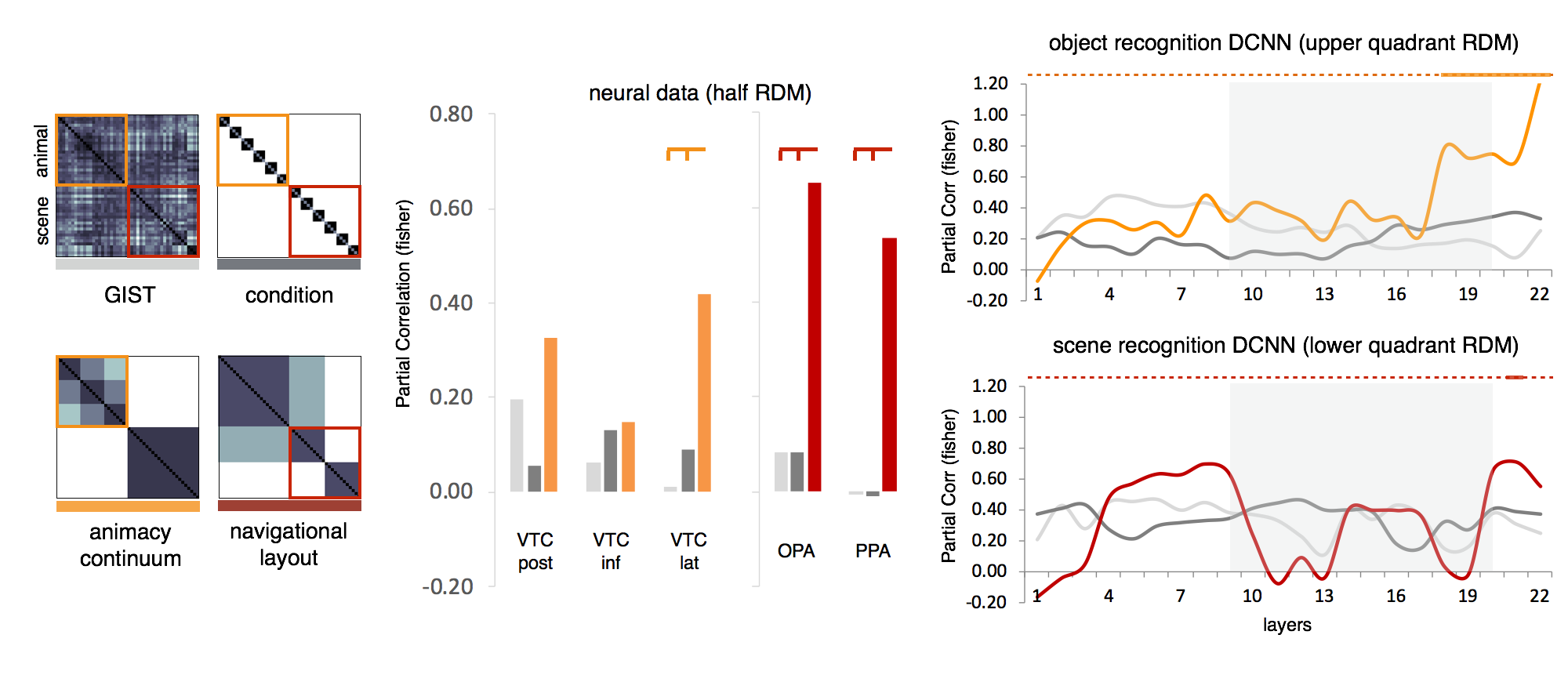


***S3 Fig. Domain-specific spaces tested within-domain data.*** *The RSA results for the 4 models (GIST, condition, animacy continuum, navigational layout) are shown for group-averaged brain (left) data and DCNNs (right). The same DCNN architecture (GoogLeNet) was trained either on object recognition (ImageNet), or scene recognition (Scene 365). For comparison with the first RSA analysis (see Fig 3A), gray shaded areas indicate the network’s layers in which the domain model significantly outperformed the remaining models. Color-coded lines on top of bar/graphs indicate the network’s layers/ROIs where each model significantly outperformed the remaining models (p < 0.001) computed with pairwise permutations tests (10000 randomizations of stimulus labels).*

**S3 Text**

As a follow-up analysis, we also tested each domain-specific model (animacy continuum or layout model) within its specific object space (animals or scenes; Supplementary Figure 3). In other words, the animacy continuum model (only its upper quandrant) was tested using only the within-animal data (upper quadrant of the neural RDM) in animal-selective areas. In the same way, the layout model (only its lower quandrant) was tested using scene data (lower quadrant of the neural RDM) in scene-selective areas. In both analyses, we also included the two control models (GIST and condition) which were constructed based on the relevant category domain (either upper or lower quadrant of the RDM). The results confirmed the main analysis showing a significant effect for the animacy continuum in VTC anterior (z = 0.42; p < 0.001, relative to the control models), and a significant layout effect the scene areas (OPA: z = 0.65; PPA: z = 0.53; p < 0.001, relative to the control models).

The same analysis was performed on DCNNs data. In the object recognition DCNN, when only animal data was considered (24x24 upper quandrant relative to the animal data), the signifincant effect for the animacy continuum model, relative to the remaining models, emerged in the high-level layers (from layer number 18) and remained high till the final layers. In the scene recognition DCNN, when using only scene data was tested, results did not show a clear preference for the navigational layout model relative to the remaining models throughtout the DCNN’s layers. Only layer number 21 showed a significant effect for the navigational layout model relative to the remaining models. These results are in line with the main analysis performed on the full dissimilarity matrix (Figure 6).

**References**

1. Walther A, Nili H, Ejaz N, Alink A, Kriegeskorte N, Diedrichsen J. Reliability of dissimilarity measures for multi-voxel pattern analysis. NeuroImage. 2016;137:188-200. Epub 2015/12/29. doi: 10.1016/j.neuroimage.2015.12.012. PubMed PMID: 26707889.

2. Smith SM, Nichols TE. Threshold-free cluster enhancement: addressing problems of smoothing, threshold dependence and localisation in cluster inference. NeuroImage. 2009;44(1):83-98. doi: 10.1016/j.neuroimage.2008.03.061. PubMed PMID: 18501637.

3. Xia M, Wang J, He Y. BrainNet Viewer: a network visualization tool for human brain connectomics. PloS one. 2013;8(7):e68910. doi: 10.1371/journal.pone.0068910. PubMed PMID: 23861951; PubMed Central PMCID: PMC3701683.
