# Supplementary material for: The representational hierarchy in human and artificial visual systems in the presence of object-scene regularities
Source: PLoS Comput Biol. 2023 Apr 28;19(4):e1011086. doi: 10.1371/journal.pcbi.1011086 (PMC10171658; doi:10.1371/journal.pcbi.1011086)
Supplement: Attachment — Submitted filename: ResponseToReviewers_final .docx [file pcbi.1011086.s004.docx]

Dear editor,

We thank the reviewers for the constructive comments, which have been helpful in revising the manuscript and improve its readability. Below we have addressed each of the comments and indicated changes made in the manuscript. In addition, to comply with copyright requirements, we have now edited figures and substituted copyright images with royalty free images. We carefully selected these images following the same criteria used to select the original stimuli. When necessary, we note this in figures labels. The stimuli used in the experiment will be available through the Open Science Framework at https://osf.io/u59ez/

**Reviewer #1:**

This paper investigates the separate and conjunctive representations of scenes and objects in the brain and deep neural network models trained for visual object recognition. A set of four models are used to capture different hypotheses on the nature of the representation. By comparing the brain activity in different regions of the ventral temporal and parieto-frontal regions, it is shown that how each area represents objects (animate) and scenes in various ways. The rest of the paper then takes a deep dive into comparing different DNN architectures on the degree of brain-similarity in representing these two category of stimuli. In summary, similar to the brain, different DNNs also show similar representations for coding objects and scenes and their interactions, capture human-like representational biases in coding scenes and objects, but fail to replicate some aspects of the brain representation - namely "multiple object spaces".

Overall, this was an interesting and well written paper that was easy to follow. The stimulus set and the accompanying fMRI experiment were designed thoughtfully. The modeling experiments were also justified and were used to support specific claims. I however had some concern with the particular method used for investigating representational similarity and found parts of the manuscript unclear.

1) The present study uses a correlation-based RSA metric to investigate similarity between different models and the brain. It is known that correlation distance metrics are sensitive to stimulus activations, unlike Euclidean distance and its derivatives [1]. It is therefore unclear to me whether the chosen metric is the best metric for the analyses carried out throughout the manuscript and to what degree the findings are dependent on this choice of metric. Can the authors show that for example by choosing a Euclidean distance metric, the claims and findings of the paper remain valid?

A similar comment was also raised by reviewer 3. To address both recommendations, we replicated the main RSA analysis (Figure 2) using the alternative distance measures suggested by the reviewers: the Euclidean distance (suggested by reviewer 1) and the cross-validated distance (following procedure reported in Walther et al., 2016, suggested by reviewer 3). These additional analyses confirmed results observed in our main correlation-based RSA. The new figure is reported in Supplementary Figure 1, and results are reported in the main text on page 7.

We understand the recommendation to run these confirmatory analyses and agree on the relevance to report results in the main text. At the same time, we would like to note that, this test allowed us to confirm results reported in a recent paper from us (Ritchie et al. 2021) which employed different distance measures (including 1-corr, Euclidean, and Mahalanobis cross-validated distance) and found no substantial differences across the measures tested in three different data sets and different ROIs. For completeness, we note this in the main text on page 7: “*To evaluate the potential impact of metrics’ choice, we re-run the RSA with two alternative distance measures: the cross-validated Mahalanobis distance (following Walther et al. 2016) and the Euclidean distance. Results confirmed the RSA analysis performed with 1 minus correlation (Supplementary Figure 1). In line with results reported in Ritchie et al., 2021, these three metrices tend to give similar results, and discrepancies are only noted in very specific designs*”.

2) It is unclear why the subjects were instructed to "indicate to what extent each image would normally co-occur with the previous image". Could this task not bias the brain representations in regions underlying decision making like PFC to be biased towards interactive effects like those observed in the study? Why hasn't a simpler task like passive fixation used instead? The logic here was unclear to me.

Yes, the task used in the present experiment might explain effects observed in frontoparietal areas, in line with the literature showing the role of frontoparietal areas in representing task-relevant information (e.g., Varizi-Pashkam and Xu, 2017; Bracci et al., 2017). In fact, this was the reason why we opted for this task: we wanted to include all stages through the brain computational hierarchy from task-independent to task-dependent areas. Ultimately, if task-dependent frontoparietal areas show object-scene co-occurrence representations, it is even more surprising that DNNs trained on object recognition alone can learn this high-level conceptual and/or task-relevant representation.

In the original text we mentioned this in Figure 1: “*The ROIs included in the brain RSA analysis included visual areas (for their relevance in object recognition) and frontoparietal areas (for their relevance in goal-directed behavior)…”.* To further clarify this point, we have now added the following sentences in the results and the discussion:

*-Results (page 6): “This task allows us to investigate representations in both task-independent and task-dependent areas (Varizi-Pashkam and Xu, 2017; Bracci et al., 2017)”.*

*-Results (page 6): ”We investigated the representational structure along the hierarchy of visual regions from the primary visual cortex over regions involved in object and scene perception up to regions in the frontoparietal cortex that encode flexible goal-directed representations”.*

*-Discussion (page 16): “Our study points in this direction, showing that, ultimately, all such interaction effects ride on top of a fundamental division of labor for animal versus scene properties, in those areas supporting flexible goal-directed representations (Varizi-Pashkam and Xu, 2017; Bracci et al., 2017).”*

3) It is unclear what "animal-selective areas" was referring to. Are all VT regions considered animal-selective?

We agree, this point needs further clarification; whereas “scene-selective” is standard terminology, “animal-selective” is not. We chose this latter terminology for the following reason. *“We know that medial VTC responds more to inanimate objects including scenes, whereas the lateral VTC responds more to animate objects including faces, bodies and animals more in general. For this reason, in a separated localizer, we defined animal- and scene-selective areas based on the direct contrast between these two conditions (animals versus scenes; scenes versus animals). This contrast successfully highlighted animal-selective areas in lateral and ventral VTC and scene-selective areas in parahippocampal gyrus (PPA), retrosplenial cortex (RSC), and transverse occipital sulcus (TOS)”.* We have now clarified this point and this contrast in the methods on page 20.

4) In figure 3, the ordering of brain ROIs in Fig. 3B implicitly suggests a particular hierarchy between them and lack thereof in the models. However, the hierarchy of these brain areas are not as strictly linear and thus this part of the figure may be misleading.

We thought about this possible issue. The reason to include Figure 3B is to provide a visualization where we are able to show (1) how the relation among models changes in the data, and at the same time, (2) how the representational content changes in both brain and DCNNs data. We understand that for brain data, by choosing a specific ROI order in the correlation matrix, we might induce the reader to think of a specific hierarchy among the ROIs. To address the reviewer’s concern, we now clarify this point in Figure 3. “*The order in which ROIs are shown does not imply a strict correspondence with the computational hierarchy in the brain*”. We hope this clarifies our intents and avoids any implicit assumption of hierarchical organization aside from the obvious assumption that computations in V1 come before VTC or that computations in posterior VTC come before anterior VTC or PFC.

5) In Figure 4, resnet50 model does not seem to follow one of the claims associated with this figure: "representational correspondence with frontoparietal areas increases and remains high till the final processing. This effect was observed regardless of DCNNs architecture.". This divergence should be discussed in text.

Thanks for pointing this out, we acknowledge that the wording in the abovementioned sentence might have been misleading. We have now edited this sentence*. “Results from this analysis confirm results reported in Figure 3 showing that representations in ventral stream domain-specific areas (animal- and scene-selective areas) are best captured by mid layers as opposed to the latest layers. Conversely, representational correspondence with frontoparietal areas does not differ between mid and top layers. This effect was observed regardless of DCNNs architecture”.* With this sentence, we wanted to highlight that whereas representations in VTC are best explained by DCNNs mid layers, frontoparietal areas instead do not show this difference: the effect observed in mid layers is maintained till the final layers.

However, note that, following suggestion from reviewer 3, we have now moved Figure 4 and the related (updated) text to supplementary materials as Suppl. Fig. 2.

6) The notion of "multiple object spaces" was somewhat unclear to me, especially in the context of DNNs. It is known that these models contain category-specific kernels that are selective for particular objects or object parts. The last section of the results seems to claim that DNNs lack "rich domain-specific object spaces". I found this claim to be contradicting more than several other works highlighting the richness of these representations e.g., see [2, 3]. Perhaps what is meant to be claimed is specific to the representational of navigational layout? If yes, this point needs to be better clarified. Even so, can this failure to find similar detailed distinction across the scene stimuli be due to the high dimensionality of these networks and the limitations of the method (RSA) used?

Thanks for pointing this out. We understand that this choice of terminology needs further clarifications also based on your comment number 9, which seems to point to a similar issue of terminology. For this reason, we address both remarks in what follows.

As the reviewer pointed out, DCNNs develop unique internal representations for different object categories (e.g., faces, cars etc), and their features (e.g., eyes for faces). These representations/features might be very “rich” already and are the result of a specific task DCNNs are trained on (e.g., object recognition). Instead, with the term domain-specific “rich/multiple” object spaces we refer to the evidence that a single computational goal (e.g., object recognition) might be unable to explain the representational content across multiple domain-specific spaces. This rich dimensionality is likely related to the need of our brain to employ different representations for different behavioural needs. One example is the evidence that in scene selective areas, the degree of navigational layout well characterizes its representational content which is relevant for navigation (e.g., Kravitz et al., 2011). Based on this evidence, what we test in the last chapter, is the ability of DCNNs, trained to recognize different object categories (scene recognition and object recognition), to capture domain-specific object spaces that might emerge as a consequence of computations, beyond object recognition. Specifically, we know that the dimension of “animacy continuum” and “navigational layout” well characterize the object space in animal- and scene-selective areas, respectively.

We hope that this clarifies the meaning of “rich/multiple object spaces” used in our manuscript. To avoid possible confusions, we have now added the following text in the results and discussion sections:

- Results (page 12): “*This rich dimensionality can support the need of our brain to employ different representations for different behavioural needs (Bracci and Op de Beeck, 2023). For instance, in scene selective areas, the degree of navigational layout well characterizes its representational content which is relevant for navigation (e.g., Kravitz et al., 2011; Park et al., 2011). In a similar fashion, in animal selective areas, the degree of animacy (Connolly et al., 2012; Sha et al., 2015), and the animal-specific features (Bracci et al., 2019; Ritchie et al., 2021) might be relevant to support social-related computations. In our results, DCNN’s mid-layers show domain division for animals and scenes (Fig. 3A), but does this division embed rich domain-specific object spaces like those observed in the human visual cortex?”*

- Discussion (page 17): *“Although DCNNs might implement different computational strategies they do still develop “rich” internal representations for different object categories (e.g., faces, objects; Dobs et al., 2022; Murty et al., 2021), and their features (e.g., eyes for faces; Serre, 2019). These representations/features are the result of specific tasks DCNNs are trained on (e.g., object recognition)”.*

As for the second point, yes, any conclusion made in the last result chapter refers to the scene domain and the navigational layout space. In our results session, we do not make claims beyond the categories tested. In the discussion, instead, we speculate that a single computational goal such as object recognition might not be sufficient to explain the dimensionality-rich domain-specific spaces observed in human VTC, also invoking evidence in the literature beyond our present study.

Finally, as for your last remark, in the results (page 14), we have acknowledged that “*…the methods employed here allow us exploring the dominant dimensions that diverge/converge across brain and DCNNs data. Alternative methods (e.g., Storrs et al., 2021) might be more sensitive to detect less preponderant dimensions yet present in the data”.*

7) Related to the previous point, it was unclear how the "navigational layout" in Fig7A produced. There are 4 columns that presumably correspond to 4 of the scene stimuli (?).

Reviewer 2 made a similar comment, which shows that the description of this model needs clarifications. We now provide a detailed description of how the models were created and moved the extended text to the methods section on page 21:

*“The navigational layout model captures the degree to which scene layout provides relevant information for scene navigation. 50% of scene images (jungle forests, ice landscapes, and beaches) contain high degree of layout information. These images are those associated with large animals (e.g., gorilla) and can be considered typical scenes that convey enough information about scene navigation. On the contrary, the remaining images contain little layout information (e.g., leaves, anemoni, and tree brunches). In other words, these images represent zoom-in scenes that from a human point of view provide little information to support navigation. Based on this consideration, the navigational layout model includes a binary distinction between these 2 sets of backgrounds. The backgrounds with low-navigational layout properties are also characterised by object-like properties, as justified by their labels (e.g., ‘leaves’), which we also incorporated in the model by a partial similarity between these backgrounds and the animal images”.*

Note that in our original text, we also reported this distinction in Figure 1: *“As for the pictures of scenes, 3 of the backgrounds are characterized by rich navigational properties where there is no object in focus in the middle of the image: seashores, ice landscapes, and jungle forests. The other 3 backgrounds are object-like scenes with little navigational layout properties: anemones, leaves, and tree branches”.*

8) Line 520: it is claimed that "object and scene information is more entangled than what we observe in the human visual system". The evidence supporting this statement is unclear to me. Please add appropriate references and discussion.

The term “more entangled” specifically refers to representations in the final DCNNs layers. In other words, the final DCNNs layers represent both the animal and its corresponding scene. On the contrary, animals and scenes are represented in separated areas in VTC. We revised the text and ensured that we always contextualize the term “more entangled” by mentioning its specificity to the DCNNs final processing layers.

The following sentences have been edited in the text:

-on page 16 (line 642): *“On the contrary, the object recognition DCNNs tested here, which are widely used in computational vision, in their final layers, do integrate background information in their learnt object space”.*

-on page 16 (line 648): *“…DCNNs in their final layers, learn representations in which object and scene information is more entangled than what we observe in the human visual cortex. In other words, the final DCNNs layers, represent both the animal and its associated scene (e.g., polar bear and ice landscape), whereas VTC represents animals and scenes in separated areas.”*

9) Also line 566-568: it is claimed that machine vision lacks "rich and diverse object spaces". It is unclear in what ways this feature is missing in current DNNs

We believe this comment relates to the one made above (see response to comment number 6). We hope that our response addressed the reviewer’s doubts and ensured that we do not claim that DCNNs miss relevant object/category features necessary to support object recognition.

10) I'm also somewhat concerned with the sufficiency of the dataset used to train the DCNN models for Fig 6. In the methods section, it is mentioned that a total of 10,500 stimuli were used to train the DNN. Considering that there are around 60 million parameters in this model, the dataset of this size sounds way too small and I'm worried about the findings from this experiment. It would be helpful to see a plot of training and validation losses for these experiments as well as examples of some of these images used for training/validation.

We agree that 10k is a small dataset example. However, it is also a relatively simple task, with 6 categories. The number of examples per category is larger than in the typical ImageNet dataset with 1000 categories and a total of 1,2 million images. In addition, we included image augmentation to improve generalization. Plots of Training and Validation accuracy show that there is some overfitting to the training images but still there is a clear improvement of performance also on the validation set (see below). It is appropriate to explicitly mention the caveat that here we are not aiming to come up with a state-of-the-art general image classification model, which is now specified as follows in the Methods section (page 22): “*Note that with only 6 image classes with a total of 10500 training images we are not claiming that the resulting DCNN models will be usable as state-of-the-art models in image classification or as reference models to compare with neural representations. Instead, these models are merely developed to provide a proof-of-principle showing how strong the impact of scene-object congruency in a training set that is controlled in terms of this congruency, in contrast to typical training sets such as ImageNet.*”.

For the 0% congruency (object occurring in a random background) and 100% congruency (object occurring always in the same background) conditions, for all 5 models we report, (A) the plots for the training/validation accuracy, and (B) examples of images for one category (animal: cat; scene: desert). Please note that images used in the training/validation set differed from images used in the test set. In the training/validation set we used images of objects with neutral background and backgrounds without objects, as shown in C.

11) typos: line 230, what -> which; line 287: in -> and; 412: an -> a

Thanks: we corrected the typos.

1. Walther, A. et al. Neuroimage 137, 188–200 (2016).
2. Cohen, U., Chung, S.Y., Lee, D.D. & Sompolinsky, H. Nat. Commun. 11, 1–13 (2020).
3. Murty, N. A. R., Bashivan, P., Abate, A., DiCarlo, J, and Kanwisher, N. Nat. Commun. (2021).doi:10.1038/s41467-021-25409-6

We have now included the suggested literature.

**Reviewer #2:**

In Bracci et al’s paper, the authors investigated the representation of the animals and their living environmental images in the human brain with fMRI, as well as the DCNN. They not only found the areas that represent the within-domain, but also the ones representing the across-domain, which indicates the co-occurrence of the animals and their living environment. The Similar representations of the within-domain and across-domain were also observed in the DCNN and the latter was dependent on the training image-sets. In addition, they also tested the animal continuum model and navigation layout model in human brain and DCNN. They confirmed that the animal continuum model was represented in both, while the navigation layout model only existed in the human brain.

In general, I think the whole experiment is well designed and some of the results are very interesting. The whole paper is well written and clear. However, I still have some comments/questions:

1) I am not sure I understand navigation spatial layout RDM model in Fig. 7A. For the animal continuum model, I understand that the authors consider the natural scene images as the non-animal images. But for the navigation spatial layout RDM, they consider the first two (or three?) groups of scene images are similar to animal images, but have zero similarity with the other scene images. How do the authors determine such RDM structure? Also for the animal continuum model, it is highly similar to the within-domain model structure. So, given the previous results, it is not surprising that they can find the animal continuum representation in the brain and DCNN. They might consider testing the animal continuum model only with animal images. The same analysis could be also applied with the spatial layout model.

Thank you for the comment. As for the first point, reviewer 1 made a similar observation. Please refer to the response to reviewer 1, comment 7. To clarify the models’ description, we have now added a new section in the methods on page 21. We hope that this clarifies.

*“The navigational layout model captures the degree to which scene layout provides relevant information for scene navigation. 50% of scene images (jungle forests, ice landscapes, and beaches) contain high degree of layout information. These images are those associated with large animals (e.g., gorilla) and can be considered typical scenes that convey enough information about scene navigation. On the contrary, the remaining images contain little layout information (e.g., leaves, anemoni, and tree brunches). In other words, these images represent zoom-in scenes that from a human point of view provide little information to support navigation. Based on this consideration, the navigational layout model includes a binary distinction between these 2 sets of backgrounds. The backgrounds with low-navigational layout properties are also characterised by object-like properties, as justified by their labels (e.g., ‘leaves’), which we also incorporated in the model by a partial similarity between these backgrounds and the animal images”.*

In our original text, we also reported this distinction in Figure 1: *“As for the pictures of scenes, 3 of the backgrounds are characterized by rich navigational properties where there is no object in focus in the middle of the image: seashores, ice landscapes, and jungle forests. The other 3 backgrounds are object-like scenes with little navigational layout properties: anemones, leaves, and tree branches”.*

As for the second point, we have now considered your suggestion to test each model within its specific category domain data (animacy continuum for animal images; navigational layout model for scene images). Results confirm our original analysis reported in Figure 6. Two main results can be observed. 1) The brain data, tested separately for each category domain (animals and scenes), is best explained by the animacy continuum and the navigational layout model, respectively. This confirms results reported in Fig 6B and rules out the reviewer’s concern on the similarity between the animacy continuum model and the domain model. 2) The main results were also replicated in the DCNNs analysis. When using animal data only (upper quadrant), in the DCNN trained to recognize objects (ImageNet), we observe a significant effect for the animacy continuum model in the high-level layers. Conversely, when using scene data only, the DCNNs trained to recognize scene categories did not show a consistent effect for the navigational layout model. It only reaches significance in layer number 20, outside the layers range where domain specific object space is expected to emerge (highlighted with grey shaded area). Thus, together these follow up analyses, performed on half dissimilarity matrix, replicate results obtained when the full dissimilarity matrix was used. These results are reported in the Supplementary Figure 3 and Supplementary Text.

In addition, I think the whole analysis is Figure 7 is not that convincing, due to the small number of images and test conditions. For the animal continuum model, they only have 3 different conditions within the animal images, and two conditions within the scene images. They can test with more images with more conditions at least with DCNN.

It is a reasonable request but difficult to implement for several reasons. The dataset we tested is extremely controlled from many factors. This is why we can compare multiple models. The problem with many datasets of natural images is that many dimensions of interests are naturally correlated with each other making impossible to disentangle among them. As an example, we consider the indoor scene dataset (*A. Quattoni, and A.Torralba.* [*Recognizing Indoor Scenes*](http://people.csail.mit.edu/torralba/publications/indoor.pdf)*. IEEE Conference on Computer Vision and Pattern Recognition (CVPR), 2009*). The database contains 67 Indoor categories, and a total of 15620 images. Some examples of scenes are airport, auditorium, concert hall, bathroom, closet, or elevator. It is quite obvious how scene category (e.g., auditorium versus closet) is totally correlated with the degree of navigational layout information present in the scene (high versus low). Also, there is a large amount of confounding (partial congruency) between the scenes and the objects in them. In other words, it might be just impossible to disentangle the relevant dimensions unless creating an ad-hoc stimulus set which will nevertheless be way too small relative to the standard datasets available in computer vision. For this reason, we believe that, to complement results based on big data sets, the need for well controlled (but inevitably small) data sets is of great added value to test cognitive/psychological theories/hypotheses.

Nevertheless, we agree with the reviewer, it is import of acknowledging the possible limitation related to the size of the dataset. We note the cons but also pros of small well controlled image sets in the discussion section on page 17.

*“For our DCNNs analyses, we employed a relatively small dataset in comparison with dataset available in computer vision. We acknowledge that this might be a limitation but at the same time, the dataset we tested has the advantage to be carefully controlled for many factors. This is why we can compare multiple competing models. The problem with many datasets of natural images is that many dimensions of interests are naturally correlated. Therefore, in many cases, it becomes difficult to disentangle the relevant dimensions unless creating an ad-hoc stimulus set which despite its size limitation, allows to tackle specific experimental hypotheses. We therefore believe that to complement results based on big data sets, the need for well controlled (but inevitably small) data sets is of great added value to test cognitive/psychological theories/hypotheses”*

2) In Figure 2, it is very interesting to see the effect of animal-scene co-occurrence emerges in frontoparietal areas. But given the low correlation value, I am not sure whether it really reflects co-occurrence. One way to test it is to build the wrong co-occurrence models (e.g., gorilla associated with landscape, and polar bear associated with forest), and then tested the significance level between the correct co-occurrence model and wrong co-occurrence models. Such analysis can be applied with ROI analysis and search light analysis to further confirm that the frontoparietal areas are really representing the co-occurrence structure that exists in the natural world.

The co-occurrence effect in frontoparietal areas is small relative to the effects observed in visual areas but we are confident that this effect is significant for two reasons. First, in frontoparietal areas, the noise ceiling is much lower relative to VTC areas (Figure 2B). The noise ceiling indicates the highest correlation we can expect between the neural data and a model after taking into account the noise in the data. As our data show, the co-occurrence model does almost reach the noise ceiling in frontoparietal areas, which suggest that it captures a substantial part of the variance that is explainable given the signal to noise of the data. Second, along the lines of your suggestion, statistical significance for results reported in Figure 2A are tested with permutation test: *“For group-averaged results, filled bars indicate significant values against baseline (p < 0.0001) computed with permutation tests (10,000 randomizations of stimulus labels”.* Note that these permutation tests, based upon random shuffling of stimulus conditions, are actually quite similar to the suggestion of building wrong co-occurrence model (the latter would result in a small subset of all possible permutations). Third, the additional tests that we performed in response to suggestions by reviewers 1 and 3 (see comment 1, rev 1 and comment 3, rev 3) further confirm the significant effects observed in these ROIs for the co-occurrence model.

Besides these two main points, I have some other minor points:

1) In Keyword part, Line 462,659, DCCN should be DCNN.
2) In line 703, the equation for fisher transformation is wrong.

Thanks for pointing out these errors. We edited the text accordingly.

3) In Fig. 2AB, 3A 6B, 7, I don’t understand why the name for Y axis is “partial correlation”. And there is no explanation for “partial correlation” in the method part.

Thanks. We now clarify this information the methods on page 22: *“For all RSAs, we used partial correlation to account for any partial relationship between models”*.

Reviewer #3: The article consists of 3 separate data-sets, each related to the question how animal and scene perception interact. A BOLD-MRI part, a DNN part with the same stimuli, and a DNN part with an extended data-set.

The topic is timely, and the article ambitious with its scope. Papers that manipulate images to test DNNs are only starting to appear and are likely one of the keys to connect psychology with AI. In particular, the results depicted in figure 3, figure 5 and figure 6 are gorgeous! Congratulations, nice work indeed.

However, I have some worries about the design of the MRI experiment, size of the stimulus set and the overall approach used for testing RDMs.

Thank you very much for the appreciation.

Specifically:
1) what is the correlation between the condition and co-occurance model? This is less relevant for the partial correlation, but comes into play in the whole brain results of figure 2. Does co-occurence significantly differ from condition? This should either be an explicit test, or the presentation should be removed (otherwise suggestive of something that is not significant).

As for the first point, the correlation between condition and co-occurrence is 0.62. We now report this in the methods section (page 21): *“The correlations between all models are the following: (domain and GIST: r = 0.11; domain and condition: r = 0.27; co-occurrence and GIST: r = 0.16; co-occurrence and condition: r = 0.62; GIST and condition: r = 0.23)”*.

As for the second point, we understand the reviewer’s remark about the whole brain RSA results. After some consideration, we thought that these maps might be of added value for the reader. First, we consider these whole-brain results (in particularly, those reported in Figure 2) a confirmatory analysis of our ROIs-based analyses, and second, even though the direct contrasts between models did not give us enough power to reveal significant whole-brain effects, by showing these maps, we can ensure that we do not miss any other interesting effect elsewhere in the brain aside our ROIs. We carefully considered the wording used and always referred to these results in terms of confirmatory analyses. For instance, this is how we reported the results for Figure 2C: *“The whole-brain RSA confirmed these results revealing that the domain model strongly activates regions in the ventral and dorsal visual pathways, whereas the co-occurrence effect for animals and matching scenes is confined within frontoparietal areas” (on page 7).”* In addition to this, we have now added the following sentence: *“We note that, although the distribution of strongest effects in Figure 2C, suggests a shift in emphasis towards frontoparietal cortex for the co-occurrence relative to the other models, this shift did not result in significant differences in a whole-brain analysis”.* We hope that this satisfies the reviewer. However, if the reviewer would strongly suggest otherwise, we would consider removing the whole-brain maps.

2) The stimuli used in part 1 and 2 of the paper are limited in size. The conclusions that the authors want to draw on the basis of fig 3 are substantial. It should be possible to do an internal replication of these results with an additional set of stimuli. Preferable of different animals and background scene's.

As for the fMRI experiment, we used 48 extremely well-controlled images. Each stimulus is presented 32 times to each participant, in two separated fMRI sessions. We carefully controlled the stimuli to make sure that alternative interpretations could be ruled out (e.g., visual similarities; background information in the image, body shape properties of the animals (e.g., two mammals, two birds) where each animal of the pair is associated with a visually/semantically different background). In our view, for an event-related fMRI study, this is a remarkable dataset with a relatively large and carefully controlled stimulus set. We hope the reviewer agrees on this.

At the same time, we understand the reviewer’s suggestion to replicate these effects in DCNNs with a larger dataset. This is indeed why we trained a DCNN on arbitrary associations between objects and backgrounds on a set of 10500 images, as a proof of principle. By showing that these arbitrary associations are specifically induced by the degree of co-occurrence we manipulated in the training set, we confirmed the results in Figure 3. We now note this on page 11: *“These results are important to also confirm the effects observed in DCNNs (Fig 3) through a much larger stimulus set”.*

3) In general, the RDM comparison approach the authors use is somewhat dated. Would it not be better to use a cross validation approach as used by for instance Storrs et al., 2020 (Diverse deep neural networks all predict human IT well, after training and fitting), or at least crossvalidation in general? Alternatively, the authors should argue why not. This is the current state of the art and with good reason.

A similar remark was made by reviewer 1. We have addressed both suggestions above. Please see response to reviewer 1, comment 1. In brief, we followed the reviewers’ suggestion and replicate our main RSA analysis using the cross-validated distance based on Walther et. al. (2016), as well as the Euclidean distance suggested by reviewer 1. These additional analyses confirmed the results observed with the correlation-based RSA. We report the results in Supplementary Figure 1 and in the text on page 7.

4) The whole brain analysis in figure 4b are correlations, and not differences between layers, however, the results are discussed in those terms (likely the differences will turn out to be not significant).

We apologize for being misleading on this point. On page 9, we have acknowledged the exploratory nature of this analysis and moved Figure 4 to the supplementary materials (Supplementary Figure 2).

5) The second data-set is more extensive than the first data-set, however the results presented in figure 7 on navigational lay-out are likely still underpowered.

We agree that the number of stimuli on which is based Figure 7 is limited. Yet, the observed correlations in Fig. 7B (now Fig 6) are high and very significant, and large effect size is beneficial for the power.

At the same time, we understand where the comment comes from. In the Discussion (page 17), we now discuss the limitation of small size datasets but also their advantages, when they are carefully controlled for dimensions of interest.

Minor comments:
* I miss the noise ceiling for BA17 and RSC for figure 2

For BA17 and RSC the noise ceiling is present but low. We have now changed visualization to make it more visible.

* There is a smooth overlap between scenes with lots of objects and a typical background. The scenes the authors use are somewhat more than a typical textures background but certainly not complex scenes. It would be good if the authors comments on this.

We understand the remark made by the reviewer. The point is that with large animals (e.g., polar bears) the scene is a relative standard scene. Instead, with small animals (e.g., ladybug) the scene is less of a scene from a human perspective and more a texture pattern. This is why in the navigational layout model in Fig. 7 (Figure 6, in the revised manuscript), the separation between high-navigation information and low-navigation information reflects this distinction. This is a consequence of trying to balance the many dimensions included in the design: animal-scene pairs, animacy continuum, and scene navigation. We clarified this point in the methods on page 21: “*the navigational layout model captures the degree to which scene layout provides relevant information for scene navigation. 50% of scene images (jungle forests, ice landscapes, and beaches) contain high degree of layout information. These images are those associated with large animals (e.g., gorilla). and can be considered typical scenes that convey information enough about scene navigation. On the contrary, the remaining images contain little layout information (e.g., leaves, anemoni, and tree brunches). In other words, these images represent zoom-in scenes that from a human point of view provide little information to support navigation. Based on this consideration, the navigational layout model includes a binary distinction between these 2 sets of backgrounds. The backgrounds with low-navigational layout properties are also characterised by object-like properties, as justified by their labels (e.g., ‘leaves’), which we also incorporated in the model by a partial similarity between these backgrounds and the animal images”.*

* What is the added value of figure 2a above figure 2b (and 2b has proper statistics).

Although, we understand the reviewer’s comment, we decided to keep Figure 2A, to show that, despite the correlations in panel B might seem somehow small, this is due to individual variability and noise in individual data. If the reviewer agrees, we would like to keep Figure 2A, which is also relevant for a remark made by reviewer 2 about the frontoparietal ROIs. Averaging across all participants brings the correlations in frontoparietal regions to a similar size as the individual-participant data in VTC ROIs. This can be predicted from the noise ceilings in Fig. 2B (which in frontoparietal areas are lower by more or less the square root of the number of participants), as such showing how the different aspects of our data analyses are very consistent with one another.

* Yes, DPFC dips less (fig 4) at the end than OPA, but OPA is still the better model for the top layer, So what point do the authors want to make? Also, are these differences significant?

What we meant is that representations in VTC or OPA, are best explained by DCNNs mid-layers rather than the top layers. However, as also acknowledged in response to your comment number 4, given the exploratory nature of the analysis reported in Figure 4, we have now moved these results to Supplementary Figure 2.

**Have the authors made all data and (if applicable) computational code underlying the findings in their manuscript fully available?**
The [PLOS Data policy](https://journals.plos.org/ploscompbiol/s/materials-and-software-sharing) requires authors to make all data and code underlying the findings described in their manuscript fully available without restriction, with rare exception (please refer to the Data Availability Statement in the manuscript PDF file). The data and code should be provided as part of the manuscript or its supporting information, or deposited to a public repository. For example, in addition to summary statistics, the data points behind means, medians and variance measures should be available. If there are restrictions on publicly sharing data or code —e.g. participant privacy or use of data from a third party—those must be specified.

Reviewer #1: **No:** the stimulus set and statistics data will be made available through OSF. The plan for sharing the code is not discussed.

Reviewer #2: Yes

Reviewer #3: **No:** The war data is not immediately open because of ethical reasons. However, the data is on OSF, and the authors indicate that on request researchers will be added to the full data. So this should be OK.

Reviewer #1: **Yes:** Pouya Bashivan

Reviewer #2: No

Reviewer #3: **Yes:** H Steven Scholte
