# Supplementary material for: The representational hierarchy in human and artificial visual systems in the presence of object-scene regularities
Source: PLoS Comput Biol. 2023 Apr 28;19(4):e1011086. doi: 10.1371/journal.pcbi.1011086 (PMC10171658; doi:10.1371/journal.pcbi.1011086)
Supplement: Attachment — Submitted filename: rebuttal_secondRevision.docx [file pcbi.1011086.s005.docx]

Reviewer #2: I have no other further questions.

Reviewer #3: The authors have gone over the comments of myself and the other reviewers with diligence. Most of my comments were fully addressed.
For point 1: I understand that the authors would like to keep fig 2c and they have now added additional text in the paper. Would it not be fair to add: the conditions do not differ from each other in the caption of figure 2c?

Nice paper!

Thanks for the feedback. We agree with the reviewer’s suggestion. This is why we already noted this in the main text (page 7, line 215) *“We note that, although the distribution of strongest effects in Fig. 2C suggests a shift in emphasis towards frontoparietal cortex for the co-occurrence relative to the other models, this shift did not result in significant differences in a whole-brain analysis”*. We can also report a similar sentence in the caption to note that some of the maps did not differ from each other. “*Note that for some of these maps (e.g., co-occurrence vs domain), the direct contrast did not reveal a significant difference*”.

For crossing the t's, the disclosure of the data and code is not entirely clear. The MRI data can be requested from the authors? It is not clear if the analysis code will be made available (currently not). Can this be rectified?

As for the raw data, due to ethics restrictions we cannot made it openly available. Nevertheless, we can certainly share all pre-processed brain functional images (beta images), anatomical masks (ROIs) and statistical data.

As for the Matlab code, in case of manuscript acceptance, we will make it available through the Open Science Framework.

We edited the Data Availability Statement accordingly.

“Due to ethics restrictions (ethics protocol B322201630276), raw brain images cannot be made available. All pre-processed brain functional images (beta images), anatomical masks (ROIs), statistics data, and the Matlab code, will be made available through the Open Science Framework at <https://osf.io/u59ez/>.”
